# Redox Regulation of Lipid Mobilization in Adipose Tissues

**DOI:** 10.3390/antiox10071090

**Published:** 2021-07-07

**Authors:** Ursula Abou-Rjeileh, G. Andres Contreras

**Affiliations:** Department of Large Animal Clinical Sciences, College of Veterinary Medicine, Michigan State University, East Lansing, MI 48824, USA; abourje2@msu.edu

**Keywords:** lipolysis, lipogenesis, redox signaling, antioxidants, oxidative stress

## Abstract

Lipid mobilization in adipose tissues, which includes lipogenesis and lipolysis, is a paramount process in regulating systemic energy metabolism. Reactive oxygen and nitrogen species (ROS and RNS) are byproducts of cellular metabolism that exert signaling functions in several cellular processes, including lipolysis and lipogenesis. During lipolysis, the adipose tissue generates ROS and RNS and thus requires a robust antioxidant response to maintain tight regulation of redox signaling. This review will discuss the production of ROS and RNS within the adipose tissue, their role in regulating lipolysis and lipogenesis, and the implications of antioxidants on lipid mobilization.

## 1. Introduction

The adipose tissue (AT) is a specialized connective tissue that functions as the primary energy storage depot in mammals. During periods of negative energy balance, lipolysis hydrolyzes triacylglycerol (TAG) reserves in AT to release fatty acid (FA) and thus meet human and animal energy needs. On the other hand, under anabolic conditions, the AT stores energy in the form of lipids, such as FA and TAG, in a process known as lipogenesis. The regulation of FA trafficking in and out of the adipocyte (i.e., lipolysis and lipogenesis) involves metabolic and endo-, para-, and autocrine pathways that depend partly on redox signaling. 

Redox signaling is a term used to describe cell signaling pathways where free radicals, or related species, serve as chemical messengers [1]. It is a fundamental process for many cell and tissue functions. Free radicals include reactive oxygen and nitrogen species (ROS and RNS), which are potent cellular metabolism products. At low concentrations, ROS and RNS are the effectors of redox signaling, but at high concentrations harm living organisms. During lipolysis, both ROS and RNS are generated by the activation of mitochondrial and cytosolic processes in AT cellular components such as adipocytes and immune cells. To maintain redox balance, antioxidant defenses are activated. However, in conditions with intense and protracted lipolysis such as human diabetes, obesity, and metabolic stress in dairy cows, the production of ROS and RNS rapidly depletes antioxidant systems, and oxidative stress (OS) develops. OS is generally defined as an imbalance between oxidants and antioxidants [2]. More precisely, it refers to increased levels of free radicals that cause cell damage. Lipids (predominantly unsaturated FA), proteins, and DNA are targets for oxidation, nitration, halogenation, and deamination by ROS and RNS [3]. This review will discuss the role that redox signaling plays in the control of lipolysis and lipogenesis in AT and the effects of antioxidants during lipid mobilization.

## 2. ROS and RNS Sources in AT

**Figure 1:**
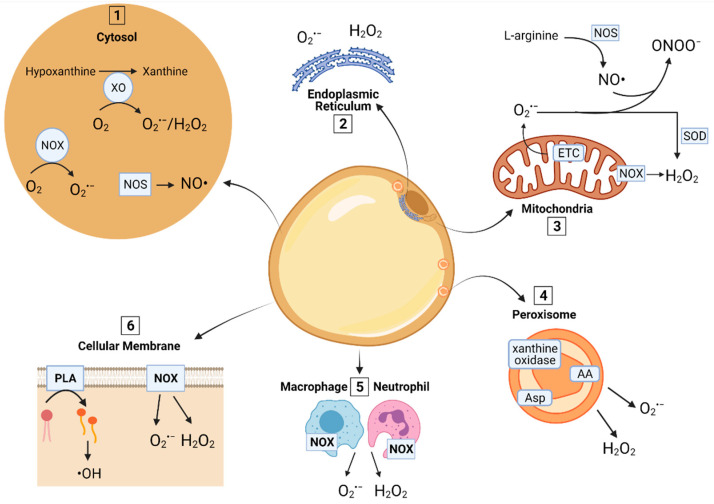
ROS and RNS sources in AT cells. (1) *Cytosol*: The oxidation of hypoxanthine to xanthine by xanthine oxidoreductase (XO) produces superoxide (O_2_^•−^) and hydrogen peroxide (H_2_O_2_). Nitric oxide synthase (NOS) produces nitric oxide (NO^•^). (2) *Endoplasmic reticulum*: Oxidative protein folding, carbohydrate addition, disulfide bond formation, and desaturation of FA generate O_2_^•−^ and H_2_O_2_. (3) *Mitochondria*: O_2_^•−^ is produced by complexes I and III of the electron transport chain (ETC). O_2_^•−^ is then converted to H_2_O_2_ by superoxide dismutase (SOD), or to peroxynitrite (ONOO^−^) in the presence of NO^•^. (4) *Peroxisomes*: O_2_^•−^ and H_2_O_2_ are produced during FA oxidation by peroxisomal enzymes such as amino acids (AA), aspartate (Asp), and xanthine oxidases. (5) *Macrophages and neutrophils*: Generate O_2_^•−^ and H_2_O_2_ by nicotinamide adenine dinucleotide phosphate oxidase (NOX) during the respiratory burst. NOX in the cytosol, cellular membrane, and mitochondria also produces O_2_^•−^ and H_2_O_2_. (6) *Cellular membrane*: Phospholipases (PLA) hydrolyze phospholipids to produce free fatty acids, which are later oxidized by cyclooxygenases and lipoxygenases, releasing hydroxyl radicals (^•^OH).

ROS is a family of free radicals, including superoxide anion (O_2_^•−^), hydrogen peroxide (H_2_O_2_), and hydroxyl radical (^•^OH). Nitrogen-containing species, referred to as RNS, include nitric oxide (NO^•^) and its derivatives peroxynitrite (ONOO^−^), nitrous anhydride, and nitrogen dioxide (NO_2_^•^) [4]. All cellular components of AT, including adipocytes, fibroblasts, endothelial cells, and adipocyte progenitors, are sources of free radicals. Within each AT cell, these sources include the mitochondria, cytosol, endoplasmic reticulum, peroxisomes, plasma membrane, and phagosomes (Figure 1; for a detailed review, readers are referred to [5]).

### 2.1. Mitochondria

The production of ROS in the mitochondria is extensively reviewed [5,6]. In short, the mitochondrial electron transport chain generates O_2_^•−^, which is the initial ROS formed, mainly at complexes I and III. Superoxide dismutase (SOD) catalyzes the dismutation (i.e., oxidation and reduction) of O_2_^•−^ to molecular oxygen and the less harmful and reactive compound H_2_O_2_. During negative energy balance-induced lipolysis, mitochondrial FA oxidation is rapidly increased, and consequently, the electron transport chain activity is enhanced. Oxidation of FA generates more O_2_^•−^ and H_2_O_2_ than that of amino acid or carbohydrate metabolites [7]. Therefore, AT is at a higher risk for developing OS during periods of negative energy balance.

### 2.2. Peroxisomes 

After the mitochondria, peroxisomes are the most abundant source of O_2_^•−^ and H_2_O_2_ in adipocytes [8]. This is because peroxisomes have relatively high FA oxidation activity (α and β) and contain active enzymes that generate free radicals such as amino acid, aspartate, and xanthine oxidases. Like in the mitochondria, as lipolysis increases, the concentration of free FA available for oxidation rises, thus enhancing free radical production. 

### 2.3. Cytosol 

Several enzymatic and non-enzymatic reactions that occur in the cytosol release free radicals. (1) The metabolism of purines and other nitrogenous bases, especially the oxidation of hypoxanthine to xanthine by xanthine oxidoreductase, produces O_2_^•−^ and H_2_O_2_ [9]. Diseases that induce hypoxia conditions in the AT, such as obesity, enhance the activity of xanthine oxidoreductase [10]. (2) Nicotinamide adenine dinucleotide phosphate oxidase (NOX) enzymes are another critical source of O_2_^•−^ and H_2_O_2_ in the cytosol and mitochondria. In adipocytes, NOX4 is abundantly expressed, and its activity releases H_2_O_2_ [11]. In fact, silencing NOX4 in rat adipocytes inhibits ROS generation during metabolic stress induced by palmitate and glucose exposure [12]. In contrast, moderate NOX4 activation by non-steroidal anti-inflammatory drugs (NSAIDs, e.g., aspirin and naproxen) reduces the production of cyclic adenosine monophosphate (cAMP), and the activation of protein kinase A leading to lipolysis inhibition [13]. 

### 2.4. Cellular Membrane 

Major ROS generators in AT cellular membranes include enzymatic reactions by phospholipases (PLA), NOX, and non-enzymatic peroxidation of lipids. PLA are present in all AT cellular components. Different isoforms of PLA2 are abundantly expressed in adipocytes, including a specific adipose isoform AdPLA [14]. PLA hydrolyze phospholipids releasing FA. Among FA, polyunsaturated FA (PUFA) are the most abundant in cellular membranes. Once released by PLA2, PUFA are oxidized by cyclooxygenases and lipoxygenases to produce tyrosyl radicals, ^•^OH, and oxylipids, including several peroxides [15,16]. Similar to O_2_^•−^ and H_2_O_2_, ^•^OH damages intracellular proteins and lipids. 

Membrane-bound NOX enzymes are the primary source of ROS from cellular membranes. Adipocyte-specific NOX4 knockout (KO) protects the carrier mice against insulin signaling dysregulation, which is one of the pathological changes leading to AT inflammation and impaired insulin sensitivity [11]. Within the AT, both macrophages and neutrophils use NOX enzymes to generate O_2_^•−^ and H_2_O_2_ from oxygen to fuel the respiratory burst reaction that is essential for their phagocytic activity [17]. Obesity and metabolic syndrome in humans are associated with infiltration and M1 phenotype polarization of macrophages. M1 macrophages have a more effective respiratory burst than M2 cells that facilitates their phagocytic activity ([18] and reviewed in [19]). However, chronic infiltration of M1 macrophages exacerbates ROS production in AT, leading to OS. In veterinary species, similar to humans, changes in macrophage phenotype polarization are associated with ROS production and OS. We demonstrated that in cows with periparturient metabolic stress that develop hyperketonemia and displaced abomasum, AT macrophages become polarized to the M1 phenotype [20]. Cows challenged with these adverse health events exhibit OS in AT [21].

### 2.5. Endoplasmic Reticulum (ER) 

The ER in adipocytes synthesizes adipokines such as leptin and adiponectin. The structure of the latter is particularly complex as it is secreted in the form of multimers. To produce these types of proteins, adipocytes’ ER relies on oxidative protein folding and other post-translational structural modifications (e.g., carbohydrate addition and disulfide bond formation) that generate O_2_^•−^ and H_2_O_2_ [22]. The level of H_2_O_2_ is rapidly reduced by adiporedoxin, an adipocyte-specific peroxiredoxin (Prx) [23]. The adipocyte ER is very sensitive to changes in its redox status, and when ER stress develops due to OS, secretion of adiponectin and other adipokines is suppressed. An additional source of ROS in the ER is the desaturation of FA. This process involves the action of desaturases (e.g., stearoyl-CoA desaturase-1) and cytochrome b_5_ that generate O_2_^•−^ as a byproduct (reviewed extensively in [24]). During lipolysis, increased availability of saturated FA, substrates for desaturation reactions, may drive ROS production by desaturases. 

### 2.6. Production of RNS 

Nitric oxide synthases (NOS) convert L-arginine to NO^•^ and L-citrulline [5]. There are three known isoforms of NOS, endothelial (eNOS), neuronal (nNOS), and inducible (iNOS). Studies performed using subcutaneous human AT [25] and rat adipocytes [26] show that both eNOS and iNOS, but not nNOS, are expressed in AT and fat cells. Hence, eNOS and iNOS are responsible for the production of NO^•^ within AT. Mechanistic evidence provided by eNOS KO mice demonstrates that the absence of eNOS activity, and consequently NO^•^, limits the development of redox signaling dysregulation related disorders [27]. RNS can also interact with ROS to produce other reactive species. For example, NO^•^ reacts with O_2_^•−^ to produce ONOO^−^ [4]. Most studies evaluating the effect of NO^•^ on lipolysis and lipogenesis have used an indirect approach through NO^•^ donors, scavengers, and NOS inhibitors. This is because the interaction between NO^•^ and oxygen makes it difficult to study its isolated function. The direct effect of NO^•^ on lipid mobilization, independent of its interaction with oxygen, remains to be explored. 

## 3. Redox Signaling and Lipolysis

Within adipocytes, the process of lipolysis involves sequential hydrolysis of triglycerides (TAG). First, adipose tissue triglyceride lipase (ATGL) hydrolyses TAG into diacylglycerol (DAG) and releases a FA molecule. Hormone-sensitive lipase (HSL) hydrolyzes DAG to monoacylglycerol, which is then further broken down into FA and glycerol by monoacylglycerol lipase (reviewed in detail by [28]). The activation of ATGL and HSL is triggered by two major lipolytic pathways, classic and inflammatory, that involve several redox signaling mechanisms at different steps during the process, including cellular membrane receptors, protein kinases, and cytoplasmic enzymes. 

**Figure 2:**
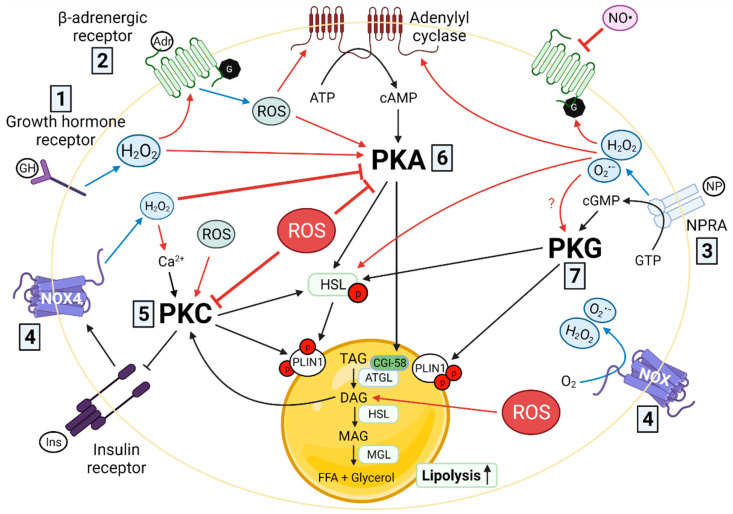
Redox signaling and lipolysis. ROS and RNS alter lipolytic pathways at different points. (1) *Growth hormone receptor*: ROS, especially H_2_O_2_, production increases upon growth hormone (GH) binding to the growth hormone receptor, consequently activating β-adrenergic receptor (βAR), adenylyl cyclase (AC), and protein kinases downstream, increasing lipolysis. (2) *β-adrenergic receptor*: Adrenalin (Adr) binds to βAR and increases the production of ROS. O_2_^•−^ and H_2_O_2_ also oxidize βAR, increasing adipocyte sensitivity to lipolysis. On the other hand, NO^•^ suppresses βAR activation. (3) *Natriuretic peptide receptor*: O_2_^•−^ and H_2_O_2_ produced upon activation of NPRA enhance the activation of βAR, AC, and cAMP synthesis, increasing lipolysis. (4) *Nicotinamide adenine dinucleotide phosphate oxidase*: NOX converts O_2_ to O_2_^•−^ and H_2_O_2_. Moreover, insulin, through the production H_2_O_2_ by NOX4, inhibits PKA activation, reducing adrenergic stimulated lipolysis. (5) *Protein kinase C* (*PKC)*: at high concentration, O_2_^•−^ and H_2_O_2_ activate PKC through the release of diacylglycerol (DAG) or may inactivate it by impairing its substrate-binding affinity. At low concentrations, ROS activate PKC by oxidizing its structural cysteine residues. H_2_O_2_ activates PKC by increasing Ca^2+^ concentrations. (6) *Protein kinase A (PKA)*: at high concentration, ROS inhibit cAMP-dependent PKA, but at low concentration, ROS prolong the activation of PKA by inhibiting the phosphatase that suppresses it. (7) *Protein kinase G (PKG)*: it is currently unknown how ROS affect PKG activity in adipocytes. Black arrows represent the classic lipolytic pathway, blue arrows represent the production of ROS/RNS, and red arrows represent the effect (activation or inhibition) of ROS/RNS.

The classic lipolytic pathway initiates by the activation of cell membrane β-adrenergic and growth hormone receptors, which in turn trigger the activity of adenylyl cyclase (AC), an enzyme that generates cAMP. The latter is a second messenger that starts intracellular signaling cascades through protein kinases. In contrast, the inflammatory lipolytic pathway is triggered through toll-like receptor 4 [29] and IL-6 cytokine receptors [30]. Lipolytic signals reach the neutral lipases (ATGL, HSL) through a series of protein phosphorylations involving protein kinases (PKA, PKC, PKG). The phosphorylation of ATGL co-activator CGI-58, perilipin 1 (PLIN1; lipid droplet coating), and HSL by protein kinases ultimately trigger lipolysis. ROS and RNS can alter the lipolytic pathways at various control points ranging from cellular membrane receptors to neutral lipase activation. However, the effect depends on the concentration, reactivity, and source of the reactive species. Below we summarize the impact of different ROS and RNS on the components of the lipolytic pathways (Figure 2).

### 3.1. Cell Membrane Receptors 

#### 3.1.1. β-Adrenergic Receptors 

G protein-coupled β-adrenergic receptors (βAR) are an integral part of the plasma membrane that bind to adrenaline and other vasoactive amines. Adipocytes express the three types of β-adrenergic receptors (β1AR, β2AR, and β3AR), and their activation induces lipolysis through PKA mediated signaling [31]. βAR signaling regulates and is regulated by redox signaling. Upon binding to adrenalin, βAR increase ROS production in a NOX and time-dependent manner [32]. At the same time, O_2_^•−^ and H_2_O_2_ can oxidize βAR by sulfenation [33]. This structural change increases the number of ligand binding sites on the βAR receptor, possibly increasing the sensitivity of adipocytes to lipolysis induced by vasoactive amines [32]. 

On the other hand, RNS, such as NO^•^ and related species, affect the lipolytic pathway by suppressing the activation of the βAR. For example, nitroglycerine, a NO^•^ donor, reduces βAR-stimulated lipolysis [34]. Likewise, *S*-nitroso-*N*-acetyl-d,l-penicillamine (SNAP), another NO^•^ donor, decreases βAR-stimulated lipolysis and cAMP production. SNAP does not affect dibutyryl cAMP (protein kinase activator), IBMX (phosphodiesterase activator), or forskolin (AC activator) stimulated lipolysis [35]. Moreover, inhibition of NO^•^ enhances βAR-stimulated lipolysis [36].

#### 3.1.2. Growth Hormone Receptor 

This class 1 cytokine receptor family member induces lipolysis in adipocytes upon binding to growth hormone (GH). Lipolysis induced by GH is particularly intense during prolonged fasting or states of negative energy balance, such as early lactation in dairy cows [37,38]. The mechanism of action for GH-induced lipolysis involves the activation of βAR 1 and 3 [39] and AC [40]. This increases ROS, including H_2_O_2_, generation at the growth hormone receptor-GH peptide interface within the cellular membrane. These ROS are ultimately responsible for the activation of the βAR, AC, and protein kinases, leading to lipolysis [41]. 

#### 3.1.3. Natriuretic Peptide Receptors 

Subtypes expressed in AT include type A (NPRA), a transmembrane protein, and NPRC, a G protein-linked receptor. These two receptors bind to natriuretic peptides (NP) and cause lipolytic effects in adipocytes [42]. The NP family includes the atrial-, brain-, and C-type NPs. Upon binding to NPRA, NPs activate guanylyl-cyclase, leading to the production of cyclic guanosine monophosphate (cGMP), which triggers the action of protein kinase G [43]. The latter phosphorylates HSL and PLIN1, leading to lipolysis activation. As with the GH receptor, activation of NPRA increases the generation of O_2_^•−^ and H_2_O_2_ in a dose and NOX2 dependent manner, possibly leading to the stimulation of βAR [44]. 

### 3.2. Adenylyl and Guanylyl Cyclases and Their Cyclic Nucleotide Products (cAMP, cGMP) 

The adenylyl cyclase/cAMP system is the target of many cell membrane receptors upon activation (e.g., βAR, NPRA). cAMP, a primary second messenger in cellular signaling, is synthesized by AC from ATP. There are at least nine subtypes of membrane-bound AC, and of those, II, IV, V, and VI are detectable in adipocytes [45]. These enzymes have 12 transmembrane domains and 2 cytoplasmic domains. Both O_2_^•−^ and H_2_O_2_ enhance the activation of membrane-bound AC and the synthesis of cAMP, triggering lipolysis [46,47]. The reduction of cAMP protects against OS by upregulating the expression of the antioxidant MnSOD. Particularly, AC5 KO mice model protects against obesity and diabetes by reducing OS in AT. This finding highlights AC’s as a critical target of ROS activity [48,49,50]. 

Guanylyl cyclase (GC) synthesizes cGMP from guanosine triphosphate. There are seven cell membrane-bound GCs. Of these, GC-A is specific for the lipolytic agent atrial NP (reviewed extensively in [51]). It is currently unknown if ROS or RNS modulate the activity of cell membrane-bound GCs. In contrast, soluble GC is activated by NO^•^ [52]. However, the lipolytic effect of soluble GC is unknown as the activity of this enzyme is compartmentalized intracellularly [53].

### 3.3. Protein Kinases

#### 3.3.1. cAMP-Dependent Protein Kinase A (PKA)

The binding of cAMP to PKA releases its catalytic subunit initiating the phosphorylation of targets including HSL, PLIN1, and CGI-58 that activate lipolysis [54]. ROS generated by the oxidizing agent diamide at high concentrations (0.5 mM) can directly inhibit PKA activity by oxidizing a highly reactive cysteine in its catalytic subunits [55]. However, at low concentrations (100 µM), diamide can inactivate the phosphatases that inhibit PKA and thus prolong the lipolytic stimulus [54]. On the other hand, low concentrations (nano to micromolar) of intracellular H_2_O_2_ inactivate PKA, and this is the mechanism by which insulin reduces adrenergic stimulated lipolysis [56]. This signaling mechanism, also termed the redox paradox, is mediated by NOX4 production of H_2_O_2_ upon insulin binding to its receptor [57]. 

#### 3.3.2. Protein Kinase C (PKC) 

This family of enzymes includes at least ten isoforms (α, β1, β2, γ, δ, ε, η, θ, D1, D2, D3). The conventional subfamily (α, β1, β2) requires DAG and Ca^2+^ for activation while the novel group (ε, η, θ) only requires DAG [58]. PKC activation induces lipolysis as this enzyme can phosphorylate HSL, perilipin, and possibly CGI-58 [59]. PKC-induced lipolysis is triggered by toll-like receptor activation, making it one of the kinases involved in the inflammatory lipolytic pathway [60]. ROS enhance or reduce PKC activity by different mechanisms. First, high concentrations of O_2_^•−^ and H_2_O_2_ can activate phospholipase C, releasing DAG from cellular membranes and activating PKC [61]. Second, H_2_O_2_ can increase intracellular concentrations of Ca^2+^ and therefore favor PKC activation [62]. Finally, O_2_^•−^ and H_2_O_2_ at low concentrations can oxidize structural cysteine residues of PKC, leading to its activation. On the other hand, at high concentrations, O_2_^•−^ and H_2_O_2_ inactivate PKC by impairing its substrate-binding affinity in a mechanism similar to the inactivation of PKA by ROS [63]. 

#### 3.3.3. cGMP-Dependent Protein Kinase G (PKG) 

There are two types of PKG, I and II. In adipocytes, PKG-I phosphorylates HSL and PLIN1 when cells are stimulated with atrial-NP [64]. Although it is currently unknown how ROS and RNS may modulate PKG-I activity in adipocytes, research in smooth muscle cells indicates that ROS and RNS activate the enzyme by oxidant-induced disulfide formation [65]. It is unclear whether or not high concentrations of ROS can inactivate PKG-I. 

### 3.4. Lipases 

#### 3.4.1. Hormone-Sensitive Lipase 

HSL is considered the rate-limiting enzyme for demand lipolysis. High and low ROS concentrations modulate the lipolytic activity of this neutral lipase. Reducing ROS concentrations with the antioxidants diphenyl iodonium (DPI), N-acetyl cysteine (NAC) and resveratrol inhibited lipolysis in human adipocytes [66]. DPI decreased both basal and forskolin (AC activator)-stimulated lipolysis. This effect is mediated by reducing the phosphorylation of an essential serine residue, Ser522, in HSL. It should be noted that all three antioxidants prevent the translocation of HSL from the cytosol to the lipid droplet under forskolin-stimulated lipolysis. Interestingly, scavenging ROS does not alter the expression of cAMP and PKA, suggesting that DPI inhibits lipolysis through direct action on HSL [66]. Aligning with this observation, Zhou, et al. [67] demonstrated that O_2_^•−^ and H_2_O_2_ can induce phosphorylation of HSL; however, their experiments did not evaluate if the mechanisms of action involved changes in the active sites of HSL.

#### 3.4.2. Adipose Tissue Triglyceride Lipase 

ATGL is the rate-limiting enzyme of basal lipolysis in adipocytes and intracellular lipolysis in other cells. ATGL activation is dependent upon the phosphorylation of its co-activator CGI-58 [68]. It is currently unknown if ROS or RNS directly modify the structures or binding properties of ATGL or CGI-58.

### 3.5. Redox Signaling Dysregulation and Lipolysis

A common pathological change in metabolic diseases is excessive and protracted lipolysis that is accompanied by AT immune cell infiltration and inflammation, cellular proliferation, and extracellular matrix changes [69,70]. Macrophages and neutrophils are the primary cells infiltrating AT. Upon activation, the respiratory burst in these cells releases ROS through a NOX-dependent process. Excessive NOX3 and NOX4 stimulation during AT inflammation enhances ROS concentrations and impairs insulin signaling in adipocytes, further intensifying lipolysis [11,71]. As AT’s free radical content increases, the organ becomes dysfunctional. For example, in obesity, a state of chronic inflammation leads to the overproduction of proinflammatory cytokines, including TNF-α, IL-1, and IL-6 in adipocytes [72]. These cytokines promote lipolysis and decrease insulin sensitivity, resulting in AT dysfunction and systemic metabolic disturbances. On the other hand, in obese mice, apocynin, a NOX inhibitor, reduces AT ROS levels, restores dysregulated adipokine secretion, and improves hyperlipidemia and diabetes [73]. Hence, excessive production of free radicals is likely a critical mechanism for enhanced and dysregulated lipolysis in metabolic diseases.

## 4. Antioxidants and Lipolysis 

**Figure 3:**
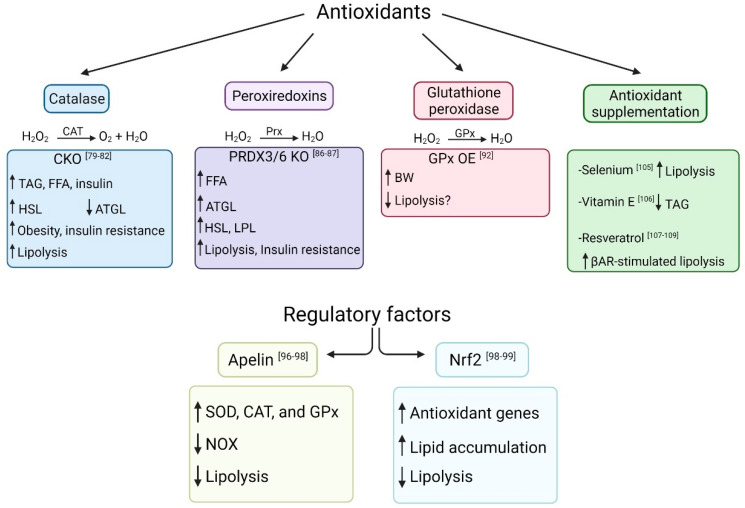
Antioxidant effect on lipolysis. *Catalase (CAT)*, an enzyme produced by peroxisomes, catalyzes the breakdown of H_2_O_2_ into O_2_ and H_2_O. CAT knockout models (CKO) fed a high-fat diet (HFD) have a higher lipolysis rate and are more susceptible to obesity and insulin resistance compared to wild-type littermates. *Peroxiredoxins (Prx)* catalyze the reduction of H_2_O_2_. Prx3/6 knockout mice (PRDX3/6 KO) have increased lipolysis and insulin resistance. *Glutathione peroxidase (GPx)* catalyzes the breakdown of H_2_O_2_ to water. GPx overexpression results in an increase in body weight (BW) possibly by decreasing lipolysis. *Selenium* promotes lipolysis during obesity. *Vitamin E* decreases plasma triacylglycerol (TAG). *Resveratrol* increases β-adrenergic receptor (βAR)-stimulated lipolysis and impairs insulin’s antilipolytic effect. *Apelin* decreases lipolysis by promoting the expression of antioxidant enzymes (superoxide dismutase (SOD), CAT, and GPx) and suppressing the expression of nicotinamide adenine dinucleotide phosphate oxidase (NOX). *Nuclear factor E2-related factor 2 (Nrf2)* increases lipid accumulation and decreases lipolysis.

As described above, ROS and RNS can enhance or limit lipolysis in adipocytes. However, dysregulated redox signaling can lead to OS when the production of oxidants exceeds the antioxidant system’s capacity (readers refer to reviews on OS in AT [74,75,76,77]). To prevent OS, antioxidant mechanisms become active during lipolysis. For instance, in dairy cows, during periods of negative energy balance, the transcription networks related to antioxidants are activated to reduce pro-lipolytic effects and OS inducers [78]. Increasing evidence shows that antioxidants play a crucial role in regulating lipid mobilization during inflammatory diseases by scavenging free radicals. The AT antioxidant system consists of enzymatic antioxidants, including catalase (CAT), peroxiredoxins (Prxs), and glutathione peroxidase (GPx). The antioxidant activity in AT is regulated at the transcription level by different cell signaling proteins and transcription factors. Non-enzymatic antioxidants such as exogenous antioxidants commonly derived from dietary sources can also enhance AT’s antioxidant capacity. We will further explain the contributions of enzymatic and non-enzymatic antioxidants to lipid mobilization in AT in the next section (summarized in Figure 3). 

### 4.1. Catalase 

CAT, an antioxidant enzyme produced by peroxisomes, catalyzes the breakdown of H_2_O_2_ into O_2_ and water. In mammals, CAT is expressed in the liver, kidney, and AT. The antioxidant capacity of CAT is severely diminished in diseases that involve AT inflammation, such as human obesity [73]. In rodent models of obesity, CAT inhibits lipolysis and prevents non-alcoholic fatty liver disease (NAFLD) by scavenging peroxisomal H_2_O_2_. The capacity of CAT to reduce lipolysis was demonstrated in CAT KO mice (CKO). These animals have heightened plasma TAG, Free FA, and insulin when fed a high-fat diet (HFD) [79]. Although not demonstrated in AT, HSL activity in the liver was enhanced while ATGL expression decreased [80]. Moreover, CAT deficient cells have more pronounced lipogenesis compared with those derived from wild-type animals [81]. Using the catalase inhibitor 3-amino-1,2,4-triazole, Nunes-Souza and colleagues [82] demonstrated that reduced CAT activity enhances lipolysis in an HSL-dependent manner. On the other hand, exogenous CAT administration eliminates the antilipolytic effect of H_2_O_2_ in the presence of epinephrine [83].

### 4.2. Peroxiredoxins 

Prxs are a family of antioxidant enzymes that catalyze the reduction of organic hydroperoxides, H_2_O_2_, and ONOO^−^ [84]. PRDX6, an enzyme belonging to the Prxs family, plays a crucial role in decreasing ROS following OS during inflammatory diseases [85]. PRDX6 KO mice fed a HFD exhibited a higher lipolysis rate reflected by increased ATGL expression and serum Free FA compared with wild-type animals. Moreover, in these mice, insulin failed to suppress AT lipolysis [86]. Likewise, PRDX3 KO murine adipocytes display greater HSL and lipoprotein lipase gene expression [87]. Taken together, these results demonstrate that the peroxiredoxins inhibit lipolysis in AT.

### 4.3. Glutathione Peroxidase 

GPx is a family of enzymatic antioxidants that reduce H_2_O_2_ to water, protecting against lipid peroxidation. It is well established that GPx serum concentration and AT expression are dysregulated during human obesity and metabolic disorders [88,89,90]. GPx alters lipid metabolism; however, its direct role on the lipolytic pathway is unknown. Some evidence suggests that GPx activity may inhibit lipolysis. The mRNA expression of GPx3 in AT is higher in lean and insulin-sensitive individuals than in those obese and insulin-resistant [91]. Overexpression of GPx1 in mice increases body weight compared with wild-type littermates [92]. This phenotype could be related to a reduction in lipolysis. Alloxan, a toxic glucose analog that generates ROS, increases lipolysis by decreasing glutathione content in adipocytes. This response is accompanied by the impairment of the redox state of the glutathione system [93].

### 4.4. Apelin 

The adipokine apelin, secreted by adipocytes in both mice and human cells, is known for its anti-obesity and anti-diabetic properties. Its levels increase in obese patients, especially in hyperinsulinemia-associated obesity [94]. Apelin binds to its G-protein coupled receptor and suppresses the production and release of ROS by promoting the expression of antioxidant enzymes (SOD, CAT, and GPx) through the ERK/AMPK pathway. Moreover, it suppresses the expression of pro-oxidant enzymes such as NOX [95]. In rat adipocytes, apelin inhibits basal lipolysis through AMPK-dependent increases in perilipin expression. At the same time, this adipokine reduces βAR-induced lipolysis by abrogating the phosphorylation of HSL at Ser-563 [96,97]. These effects are also observed in vivo, where apelin-KO mice have significantly higher serum FA and glycerol compared to wild-type mice, yet this effect is abrogated after apelin infusions [97]. To summarize, apelin decreases lipolysis by stimulating antioxidant expression. 

### 4.5. Nuclear Factor E2-Related Factor 2 (Nrf2) 

Nrf2 is a basic leucine zipper (bZIP) protein associated with the cytoplasm. When cytoplasmic ROS levels increase, Nrf2 translocates to the nucleus and initiates the transcription of various antioxidant genes [98]. Nrf2 activation appears to reduce lipolysis in AT. In 3T3-L1 adipocytes, Nrf2 knockdown reduces H_2_O_2_-induced lipid accumulation. Nrf2 KO mice also have reduced transcription of lipogenic genes and increased ATGL and HSL activity when fed chow and HFDs [99]. Nrf2 activation in mice reduced HFD-induced lipid accumulation in white AT and HFD-induced obesity [100]. 

### 4.6. Antioxidant Supplementation 

Under physiological conditions, endogenous antioxidants can prevent excessive ROS/RNS production. However, there is a continuous demand for exogenous sources such as selenium (Se) and vitamin E. These antioxidants are known to be effective in reducing OS in many human [101,102,103] and cattle [104] diseases. Se supplementation promotes adipocyte differentiation in AT; however, during obesity, it promotes lipolysis by activating the classic lipolytic pathway (PKA/HSL) in a dose-dependent manner [105]. Vitamin E supplementation improves insulin sensitivity in obese mice models and reduces plasma TAG levels [106]. Lastly, resveratrol, a naturally occurring phenolic compound, enhances lipid mobilization upon βAR activation but has no effect on basal lipolysis. At concentrations of10 µM, resveratrol increases βAR-stimulated lipolysis and impairs insulin’s antilipolytic response [107]. Similar results are observed in rat adipocytes [108] and human AT explants [109] stimulated by epinephrine. It is important to note that ROS levels were not directly measured under these conditions.

## 5. Redox Signaling and Lipogenesis

**Figure 4:**
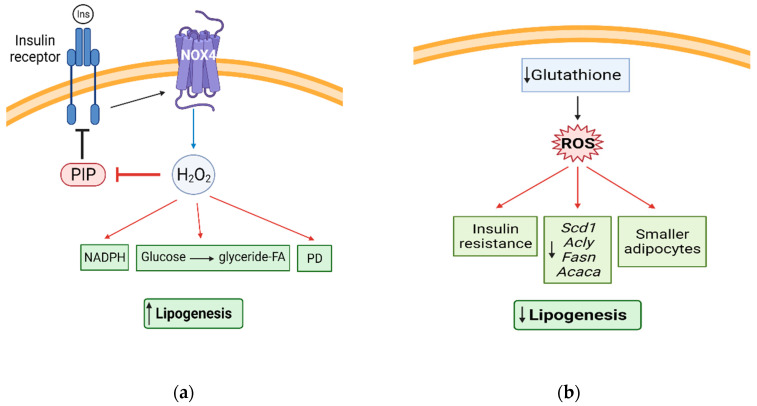
Redox signaling and lipogenesis. (**a**) H_2_O_2_ acts as a secondary messenger of insulin in adipocytes by suppressing the oxidation of protein tyrosine phosphatases (PIP), thus facilitating insulin signaling. H_2_O_2_ also increases lipogenesis by increasing NADPH and glucose incorporation into glyceride-FA, and stimulating pyruvate dehydrogenase (PD); (**b**) enhanced Fat ROS, through the depletion of glutathione in adipocytes, decreases insulin sensitivity, reduces lipogenic gene expression, and display smaller adipocytes.

Reactive species can also modulate the lipogenic pathway. Lipogenesis refers to FA and TAG synthesis, which takes place in both the liver and AT. Within AT, TAG can be hydrolyzed to release FA by lipoprotein lipase (LPL) [110]. FA then enter adipocytes through fatty acid transporters such as CD36 and fatty acid transport protein-1 (FATP1) [108]. These FA can be esterified to form TAG and stored in the lipid droplet. Alternatively, in *de novo* lipogenesis, circulating carbohydrates are converted into FA that are then used for synthesizing TAG or other lipid molecules. This process can be stimulated by insulin through *GLUT4*, which triggers glucose uptake by adipocytes [111]. Some of the rate-limiting enzymes in lipogenesis include fatty acid synthase (*Fasn*), diacylglycerol O-acyltransferase 1 (*Dgat1*), stearoyl-CoA desaturase-1 (*Scd1*), and acetyl-CoA carboxylase (*Acaca*). Many studies have shown that redox signaling modulates lipogenesis mainly through H_2_O_2_ (Figure 4). 

### 5.1. H_2_O_2_ and Lipogenesis

For decades, H_2_O_2_ has been suggested to play an essential role in cellular events, including glucose transport and uptake. More specifically, it is the second messenger of insulin in adipocytes [112]. At low concentrations, it inhibits the oxidation of protein tyrosine phosphatases, thus facilitating insulin signaling [57]. In rat adipocytes, H_2_O_2_ (0.15–0.5 mM) was shown to stimulate glucose carbon incorporation into glyceride-FA [113]. H_2_O_2_ increases lipogenesis by enhancing substrate transport and NADPH along with stimulating pyruvate dehydrogenase. This effect is abolished in the presence of CAT [83]. The concentration of H_2_O_2_ is a major factor in determining whether it enhances or suppresses lipogenesis in AT since OS has been shown to cause insulin resistance and impair lipolysis inhibition [114,115]. 

### 5.2. FA and TAG Synthesis 

ROS increase lipid synthesis by promoting glucose use to synthesize lipids. Increasing ROS production with acetoacetate (Acoc, 20 mM) activates *de novo* lipogenesis in human adipocytes by enhancing glucose conversion to FA. Acoc also induces lipolysis, but the lipolytic rate does not exceed the rate of lipogenesis [116]. Treatment of mature 3T3-L1 adipocytes with the natural antioxidant in lyophilized cranberries decreases ROS levels by 29.3% and lipid accumulation in a dose-dependent manner. This is also accompanied by an increase in basal lipolysis [117]. 

The generation of mice with genetically manipulated ROS in adipocytes allows us to understand better the role of ROS in lipid synthesis. Through the overexpression of CAT and SOD1, Fat ROS-eliminated mice display enhanced insulin sensitivity and AT expansion. *De novo* lipogenesis in WAT from these mice is enhanced and is associated with increased expression of FA-synthesizing genes (*Acly, Scd1, Fasn*, and *Acaca)*. On the contrary, mice with enhanced content of ROS in adipose depots, through the depletion of adipocyte glutathione, exhibit smaller-sized adipocytes with decreased expression of lipogenic genes (*Acly*, *Scd1*, *Fasn*, *Acaca*, and *Srebf1)*. ROS-induced downregulation of lipogenic genes appears to be mediated through the suppression of sterol-regulatory element-binding transcription factor 1 transcriptional activity in rat adipocytes [116]. Similarly, octanoate, a medium-chain FA, inhibits lipogenesis through the decrease of key lipogenic genes including *LPL, Fasn*, and diacylglycerol acyltransferase 2 in rat adipocytes [118]. This response may be mediated through the generation of ROS. These results suggest that ROS production in adipocytes might directly inhibit *de novo* lipogenesis. 

## 6. Redox Signaling and Dairy Cows’ Lipid Mobilization 

Similar to humans and other animal models, alterations in redox signaling, and the consequent development of OS, act as a determining factor in abnormal inflammatory responses in the AT of dairy cows, especially during the periparturient period [119,120,121]. This segment of the lactation cycle, spanning from 3 weeks before calving until 3 weeks postpartum, is characterized by intense lipolysis and limited lipogenesis. As a consequence, the AT generates vast amounts of ROS. Significant sources of ROS in periparturient cows’ AT include mitochondrial activity and the production of oxidized fatty acids, termed oxylipids. We demonstrated that lipolysis is determinant in the biosynthesis of oxylipids as it provides abundant substrates (unsaturated FA) for their biogenesis by enzymatic and non-enzymatic reactions [122]. Higher maternal ROS metabolites in blood, especially in cows with high body condition scores, are associated with greater lipolysis [123]. Moreover, enhanced energy needs for fetal growth and lactogenesis, increase mitochondrial respiration that in turn enhances O_2_^•−^ and H_2_O_2_ production. More research is needed on the activity of other major sources of ROS, such as peroxisomes and ER, and RNS in AT of periparturient cows to better direct nutritional or pharmacological interventions aimed at minimizing OS. 

As AT lipolysis intensity increases postpartum, the antioxidant defenses of AT become active. The transcription of GPx system components, including glutathione peroxidase 1 and transaldolase 1, is upregulated as well as the protein abundance of glutathione S-transferase mu 1 [124]. Other physiological conditions associated with an intense lipolytic response also trigger antioxidant defenses in dairy cows. For example, a proteomics analysis performed in dairy cows with heat stress identified the Nrf2 OS response components as one of the top canonical pathways upregulated compared to control cows [78]. A comprehensive characterization of OS during the periparturient period or health events in AT of dairy cows is currently lacking. However, there is evidence for the presence of OS in AT of these cows as we detected isoprostanes, the gold standard OS biomarker, in AT during the first three weeks after calving [125]. 

## 7. Conclusions and Future Prospective 

To summarize, ROS/RNS regulate lipid mobilization in AT by modulating different lipolysis and lipogenic signaling pathways. Uncontrolled production of ROS favors lipolysis. However, one should not generalize about the direct effect of ROS and RNS on lipid mobilization since each species is unique in its function. Free radical actions will depend on the reactive species, its origin/source, concentration, and length of exposure. Likewise, AT antioxidant mechanisms function differently as they act on distinct ROS/RNS. More research is needed to determine the effect of specific antioxidants to optimize their clinical use and as nutritional supplements. Moreover, direct measurement of particular ROS or RNS, such as O_2_^•−^ and NO^•^, is limited and complex. Therefore improving the sensitivity and specificity of ROS/RNS detection in AT is essential to expand our understanding of redox signaling and OS development. 

## Abbreviations

ROS	Reactive oxygen species
RNS	Reactive nitrogen species
AT	Adipose tissue
TAG	Triacylglycerol
FA	Fatty acid
OS	Oxidative stress
O_2_^•−^	Superoxide anion
H_2_O_2_	Hydrogen peroxide
^•^OH	Hydroxyl radical
NO^•^	Nitric oxide
ONOO^−^	Peroxynitrite
NO_2_^•^	Nitrogen dioxide
NOX	Nicotinamide adenine dinucleotide phosphate oxidase
NOS	Nitric oxide synthases
ATGL	Adipose tissue triglyceride lipase
HSL	Hormone sensitive lipase
DAG	Diacylglycerol
AC	Adenylyl cyclase
βAR	β-adrenergic receptor
PKA	Protein kinase A
PKC	Protein kinase C
PKG	Protein kinase G
Prx	Peroxiredoxin
CAT	Catalase
GPx	Glutathione peroxidase
SOD	Superoxide dismutase
LPL	Lipoprotein lipase
